# Editorial: Socio-psychological perspectives on collective behavior and social movements

**DOI:** 10.3389/fpsyg.2023.1266567

**Published:** 2023-08-22

**Authors:** Juan Carlos Oyanedel, Agustin Espinosa, Huseyin Çakal, Dario Paez

**Affiliations:** ^1^Faculty of Social Sciences and Education, Andres Bello University, Santiago, Chile; ^2^Department of Psychology, Pontifical Catholic University of Peru, Lima, Peru; ^3^School of Psychology, Keele University, Newcastle-under-Lyme, United Kingdom

**Keywords:** social psychology, wellbeing, social movements, collective behavior, social identity

This Research Topic seeks to examine antecedents and effects of participation in collective behavior (CB) and social movements (SM). The scholars participating on it come from different regions of the world and examine different scenarios of political action and reasons for CB. Let's start for da Costa et al., whose systematic review helps outlining the field. The authors conclude that participation in CB and SMs was associated with conflict over resource allocation, intergroup dynamics, and realistic threats. Karataş et al. paper describes a reliable measure to study positive and negative intergroup contact between minority and majority ethnic youth, a relevant factor for understanding SM. Estela-Delgado et al. examine the role of economic crises, an important factor in SM, showing that personal wellbeing is positively associated with financial wellbeing, which, in turn, is negatively associated with financial threats.

da Costa et al. also found that participation in CB and SM was explained by relative deprivation (RD), identity and collective efficacy, marking another path to explore their relationship. Peng and Wu illustrate the importance of RD for wellbeing, showing how in China relative individual income deprivation decreases sleep duration, mediated by a decrease in social trust. Wu et al. show how differential leadership, mediated by RD, reinforces deviant innovation behaviors—a phenomenon close to the mobilization of social change.

da Costa et al. systematic review also showed that affective relative deprivation and emotions like anger favor participation in SM, as well as ideological factors such as moral commitment and perceived threat to moral values and disagreement with system justification beliefs. Villagrán et al. findings support the importance of emotions in SM, in the context of the 2019 social outbreaks in Chile and Ecuador: people who are more interested in politics are more likely to experience anger with the social situation, and people who showed greater concern about the impacts of the COVID-19 pandemic and feel anger report higher conventional and online participation in SM. Analyzing the social outbreak in Chile, Hatibovic et al., confirm that emotions like anger toward police and positive emotions toward demonstrators predict the disposition to participate in conventional and unconventional CB. Carrasco Paillamilla and Disi Pavlic study on social outbreak in Chile found that participation in demonstrations and proximity to violent protests have ideological effects, being associated with the perception that security forces frequently violated human rights during the outburst. Moyano-Díaz et al. examine a state of anomie as an explanatory factor for the Chilean social outbreak, since a perception of high anomie dominated. The perception of the rupture of the social fabric was related to believing that the governments of both the right and left are impotent to fight crime. Leadership breakdown was negatively related to political interest. Espinosa et al. examine similar phenomena in a Peruvian sample. Attitudes toward populist social movements were related to cynicism or political mistrust, and negatively to the perception of change in the political system, suggesting that populist attitudes arise in the context of political mistrust and express an ambivalent relationship with democracy. On the other hand, Ballesteros-Quilez et al. systematically review the literature on the squatter SM and conclude that it is based on collective actions with a political role of resistance to neoliberalism and the inequalities related to it, and of response to the needs of the communities through self-management. Kim and Lee examine how a power or control approach underlies the US capitol occupation report, which emphasizes policing deficits. They propose a more complex psychosocial approach that considers the processes of legitimacy, procedural justice, the evolution of collective identity, and negotiation can contribute to a more efficient and rational police action. Wang and Ren, in a study that examines a distal factor of SM, show that processes of social mobilization and migration reinforce cultural individualism. Pizarro et al. meta-analysis showed that the collective effervescence or perceived emotional synchrony, experienced while participating in collective gatherings, reinforces and modifies personal and collective emotions, identity and social integration, self-efficacy and individual and collective esteem, as well as the agreement with ideological values. Zabala et al. confirms the positive effect of participation on collective gatherings on social wellbeing and its maintenance through collective effervescence for at least 6–7 weeks after the event. It also shows that self-transcendence emotions like *Kama muta* are relevant during collective gatherings. Finally, Carvacho et al. show that the failure of the SM, leads the participants to increase their willingness to participate in the future, compared to non-participants, who decrease it. In another study, failure increases the perception of efficacy, in people with a history of non-conventional or non-normative participation. These results are consistent with those reported in the systematic review by da Costa et al. on the medium-term effects of participation in SM and show that their failure does not automatically lead to disempowerment of those who participate in it.

In this introduction, we also want to reflect, based on the studies examined, on why people participate in CB and SM—particularly recreational or not linked to processes that directly seek social changes. The review of the explanatory theories of SM and of the reasons for participating in collective gatherings (see da Costa et al.), as well as the theories of motivations (Sheldon et al., 2001) and of motivational purposes or values (Schwartz, 1999) suggest that participation in CB and SM can satisfy 17 different motives.

CB can satisfy needs or motives linked to physical adaptation, stimulation, hedonism, and aesthetic pleasure, as well as emotion regulation—these reasons are more relevant in festivals and playful encounters, although the last three probably plays a role in the CBs linked to SM. Material motives or incentives can play a role in some cases. Olson (1971), in his classic text on why people did not participate in SM, argued that only specific incentives led to abandoning the logic of the free rider. Studies show that this motive is not very relevant for participation in SM, although both the attainment of money and expression of wealth may play a role in ludic CB. Another set of motives is linked to empowerment (self-direction, self-efficacy and self-esteem), both individual and collective, as well as to the motivation of power and influence—this motive will be relevant for leaders and militants above all. Another group of motives is linked to self-realization, learning and creativity. Motives like search of or attribution of meaning, maintaining a tradition and social norms, as well as satisfying the need for security are also relevant. Affiliation and relational motives are also relevant for CC related to SM. Finally, motives of self-transcendence or collective motives, related to fighting to change society for the better are relevant—particularly for religious and ideological SM.

These motives highlight the role that social identity, in its different configurations, has for both CB and particularly for SM participation. While relative deprivation continues to be a relevant factor for identity creation and mobilization, elements such as recognition by the authorities and the perception of their efficacy can also affect the self-image of actors and their willingness to engage either in CB or SM.

Finally, it is also important to see what happens the other way around, and to examine how successful participation in CB and SM can reinforce all aspects of wellbeing, as is shown in Table 1 (Oyanedel and Paez, 2021).

**Table 1:** Potential motives related to participation in successful CB and positive effects on wellbeing.

**Psychological needs (Sheldon et al., 2001)**	**• Values • motivational goals (Schwartz, 1999)**	**Reasons or motives to participate in collective leisure and recreational gatherings (da Costa et al.)**	**Motivation for participation in all types of CB**	**Dimensions subjective and psychological wellbeing (Oyanedel and Paez, 2021)**
Thriving		Thriving: improve physical fitness	Thriving	Vitality
Pleasure	Hedonism	Hedonic: have fun	Pleasure	Emotional wellbeing
	Stimulation	Stimulation: obtaining excitement	Stimulation	
		Aesthetic: enjoy scenery, nature	Aesthetic	
		Affect regulation: rest relax; recover from stress	Coping and change emotions	
Money			Instrumental for individual	
Self determination Competence	Self-direction Achievement	Competence: developing/evaluating their competences; increasing self-confidence	Competence	Mastery
Autonomy Self determination	Autonomy Self-direction	Autonomy: satisfy need for independence; freedom make your decisions;	Autonomy	Autonomy
Self-esteem		Self-esteem: creating a good impression in front of other people	Self-esteem	Acceptance
Popularity influence	Power	Power: experiencing leadership; be able to control	Power	
Self-actualization		Self-actualization: develop values; grow spiritually	Self- actualization	Personal growth
		Learning, expanding knowledge	Knowledge	
		Creativity, do creative things; Live new experiences	Creativity	
Security	Security	Security: to be near considerate people, risk reduction and avoidance	Security	
	Tradition Conformism		Meaning attribution	Purpose in life
Relatedness	Benevolence	Affiliation: do things with family and acquaintances; being with people who do the same things and have the same values; social sharing	Relatedness	Positive relationships with others
	Universalism	Express collective identity relive the history of the community	Instrumental to change society	Self-transcendence

## Author contributions

JO: Conceptualization, Funding acquisition, Writing—original draft, Writing—review and editing. AE: Conceptualization, Validation, Writing—review and editing. HÇ: Conceptualization, Validation, Writing—review and editing. DP: Conceptualization, Writing—original draft, Writing—review and editing.
